# Imaging Invasion: Micro-CT imaging of adamantinomatous craniopharyngioma highlights cell type specific spatial relationships of tissue invasion

**DOI:** 10.1186/s40478-016-0321-8

**Published:** 2016-06-03

**Authors:** John R. Apps, J. Ciaran Hutchinson, Owen J. Arthurs, Alex Virasami, Abhijit Joshi, Berit Zeller-Plumhoff, Dale Moulding, Thomas S. Jacques, Neil J. Sebire, Juan Pedro Martinez-Barbera

**Affiliations:** Institute of Child Health, University College London, London, UK; Great Ormond Street Hospital, London, UK; Department of Histopathology, Royal Victoria Infirmary, Newcastle, England; μVIS X-ray Imaging Centre, Faculty of Engineering and the Environment, University of Southampton, Southampton, UK

## Abstract

Tissue invasion and infiltration by brain tumours poses a clinical challenge, with destruction of structures leading to morbidity. We assessed whether micro-CT could be used to map tumour invasion in adamantinomatous craniopharyngioma (ACP), and whether it could delineate ACPs and their intrinsic components from surrounding tissue.

Three anonymised archival frozen ACP samples were fixed, iodinated and imaged using a micro-CT scanner prior to the use of standard histological processing and immunohistochemical techniques.

We demonstrate that micro-CT imaging can non-destructively give detailed 3D structural information of tumours in volumes with isotropic voxel sizes of 4–6 microns, which can be correlated with traditional histology and immunohistochemistry.

Such information complements classical histology by facilitating virtual slicing of the tissue in any plane and providing unique detail of the three dimensional relationships of tissue compartments.

**Electronic supplementary material:**

The online version of this article (doi:10.1186/s40478-016-0321-8) contains supplementary material, which is available to authorized users.

Tissue invasion and infiltration by brain tumours poses a clinical challenge, with destruction of eloquent structures leading to morbidity, and the inability to separate tumour and normal tissue limiting the ability to perform a complete surgical resection required for cure.

Microfocus Computed Tomography (Micro-CT) is an emerging technique developed to provide very high resolution imaging of biological specimens. Although designed for the engineering industry (for non-destructive testing of components) and used for archaeological specimens, recent use in small animal phenotyping suggests it could have a role in ex-vivo human tissue evaluation, preserving tissue integrity and allowing subsequent histological examination [1–4].

Adamantinomatous craniopharyngiomas (ACP) contain several different cellular compartments of different cellular density (including palisading epithelium, stellate reticulum, epithelial whorls/clusters and “wet keratin”) and a complex pattern of invasion, such as finger like protrusions of tumour within an often florid glial tissue reaction [5]. As Micro-CT imaging of tissues relies on differential X-ray absorption between tissue components, we assessed whether micro-CT could be used to delineate ACPs and their intrinsic components.

Three anonymised archival primary frozen ACP samples (two paediatric, one adult, Additional file 1: Table S1) were fixed in 10 % formalin and then placed in potassium tri-iodide for at least 72 hours to improve CT contrast. Images were acquired using a Nikon XTH225 ST micro-CT scanner. After imaging samples were embedded in paraffin and processed by standard protocols, including staining with Haematoxylin and Eosin and immunohistochemistry for glial fibrillary acidic protein (GFAP) and beta-catenin. The results are provided in Figs. 1 and 2 and Additional file 2: Video 1, Additional file 3: Video 2, Additional file 4: Video 3 and Additional file 5: Video 4.

**Fig. 1:**
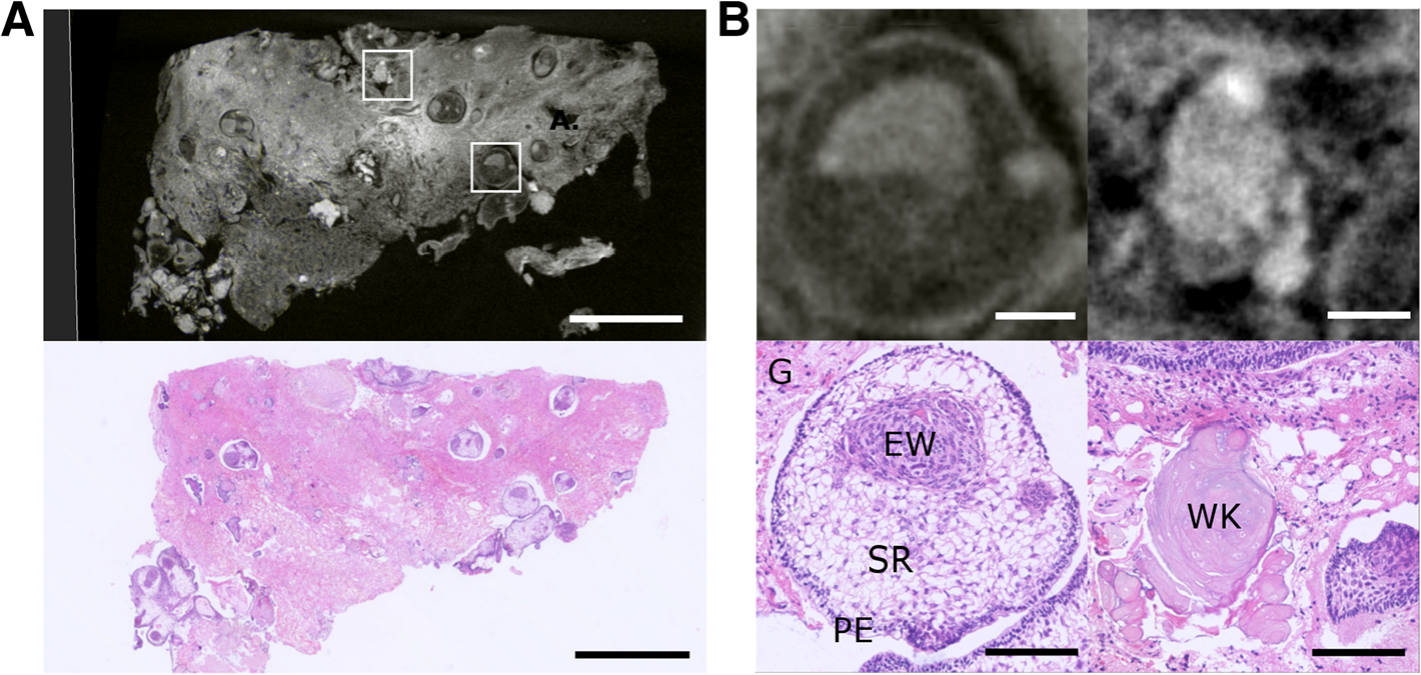
Micro-CT imaging of adamantinomatous craniopharyngioma: **a** Virtual and matched histological tissue section of ACP case 1 showing areas of tumour interspersed by reactive glial tissue. Scale bar indicates 1 mm. **b** 20x images of specific tumour compartments from boxed regions of A. The left panel shows epithelial whorls (“clusters”) within an area of tumour and the right panel shows “wet keratin” which has a higher grey value on CT imaging. Scale bars indicate 100 μm. EW = Epithelial Whorls, SR = Stellate Reticulum, PE = Palisading Epithelium, G = Reactive Glial Tissue, WK = Wet Keratin

**Fig. 2:**
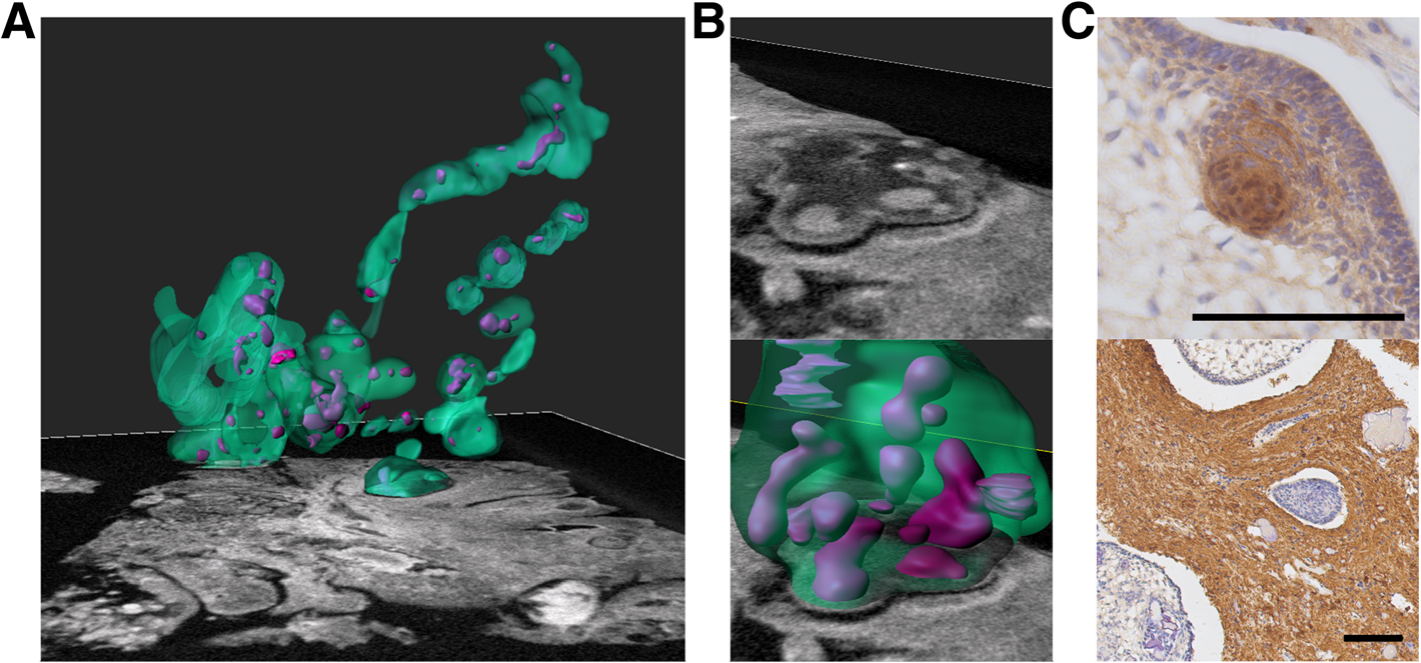
**a** Three dimensional annotation of an area of case 1. Green indicates the border of tumour demonstrating nodules and islands with some interconnections. Connections of less than 5 μm will not be well visualised at this resolution, possibly explaining discontinuities. Purple indicates epithelial whorls/clusters. **b** An area of finger-like protrusions. The upper panel shows the micro-CT image; the lower panel shows 3D annotation revealing a complex 3D structure in this region. **c** Immuno-histochemical staining of the post micro-CT samples in (A) demonstrating appropriate antigenic reactivity following iodination. Upper panel beta-catenin showing a cluster with nucleo-cytoplasmic accumulation (case 3), lower panel glial fibrillary acidic protein (GFAP) (case 1). Scale bars indicate 100 μm

We demonstrate that micro-CT imaging can non-destructively give detailed 3D structural information of tumours in volumes with isotropic voxel sizes of 4–6 μm (equivalent to a resolution of 5–7 μm when taking account of the focal spot size of 3 μm [6]) with excellent internal contrast, equivalent to that of low power histological examination (Fig. 1, Additional file 2: Video 1, Additional file 3: Video 2 and Additional file 4: Video 3). Micro-CT image slices could be correlated with their histological counterparts (Fig. 1). Such information complements classical histology by facilitating virtual slicing of the tissue in any plane and providing unique detail of the three dimensional relationships of tissue compartments (Additional file 2: Video 1, Additional file 3: Video 2 and Additional file 4: Video 3).

The majority of ACPs harbour somatic activating *CTNNB1* mutations, but surprisingly, on immuno-staining, nuclear-cytoplasmic accumulation of beta-catenin is frequently restricted to a minority of cells, often grouped into “clusters” which mostly correlate to dense epithelial whorls [5]. Evidence from mouse models suggests a key role for these clusters in tumour initiation and/or progression. Genetically engineered mouse models have demonstrated that clusters secrete a plethora of growth factors and cytokines and a murine xenograft model has revealed that clusters at the leading edge of tumour invasion may be responsible for the infiltrative behaviour [7–10]. The spatial relationship of these clusters to tumour infiltration was further explored in 3D by utilising advanced semi-automated image processing software (Imaris (Bitplane AG) and VG Studio MAX (Volume Graphics GmbH)) to extract contour lines for both tumour and clusters from the micro-CT image stacks. Differential grey values allowed tumour boundaries and clusters to be segmented from reactive glial tissue within manually determined regions. Segmentation tools merged the largest connected areas bounded by the maximum intensity of voxels within a user-defined range, creating a three dimensional model (Fig. 2a & b). This highlighted the complex relationships of tumour and reactive tissue with nodules and islands interspersed across a region of the sample. An area of apparent “finger like protrusions” was further analysed and found to be part of a relatively larger complex area of tumour tissue (Fig. 2b). Clusters were visualised predominantly at protrusions of tumour in both areas assessed, consistent with their suggested role in promoting invasion (Fig. 2a and b).

Micro-CT is a non-destructive technique that does not preclude subsequent histological processing or staining. All diagnostic features were preserved and immuno-staining successful following potassium tri-iodine staining (Fig. 2c), and we found similar imaging features following paraffin embedding (Additional file 5: Video 4).

We present the first 3D assessment of the cellular relationships involved in tumour infiltration using micro-computed tomography (Micro-CT) imaging. As pathology slowly enters the digital era techniques such as micro-CT have the potential to revolutionise the way tissues, and tumour invasion, may be visualised and understood in 3D at a resolution previously unachievable by other imaging modalities.

## Additional files

Additional file 1: Table S1. Case Details. (DOC 27.5 kb) Additional file 2: Video 1. Virtual dissection of a human adamantinomatous craniopharnygioma specimen (case 1) by micro-CT imaging. Nodules and islands of tumour are visible within reactive glial tissue. Epithelial whorls/clusters are present. Areas of “wet keratin” have higher grey level values (appear whiter). The micro-CT isotropic voxel size achieved in each case was related to the degree of magnification achieved and is 4.22 μm for this case. (WMV 191950 kb) Additional file 3: Video 2. Virtual dissection of a human adamantinomatous craniopharnygioma specimen (case 2) by micro-CT imaging. Nodules and islands of tumour are visible within reactive glial tissue. Epithelial whorls/clusters are not a prominent feature in this case. Areas of “wet keratin” have higher grey level values (appear whiter). The micro-CT isotropic voxel size achieved in each case was related to the degree of magnification achieved and is 4.5 μm for this case. (WMV 151528 kb) Additional file 4: Video 3. Virtual dissection of a human adamantinomatous craniopharnygioma specimen (case 3) by micro-CT imaging. Nodules and islands of tumour are visible within reactive glial tissue. Epithelial whorls/clusters are present. Areas of “wet keratin” have higher grey level values (appear whiter). The micro-CT isotropic voxel size achieved in each case was related to the degree of magnification achieved and is 6.8 μm for this case. (WMV 105536 kb) Additional file 5: Video 4. Virtual dissection of case 1 by micro-CT imaging showing epithelial whorls within the tumour and other features persist following embedding in paraffin. Isotropic voxel size was 5.69 μm. (WMV 79450 kb)

## Abbreviations

ACP: adamantinomatous craniopharyngioma
GFAP: glial fibrillary acidic protein
Micro-CT: Microfocus Computed Tomography
