# Supplementary material for: Imaging Invasion: Micro-CT imaging of adamantinomatous craniopharyngioma highlights cell type specific spatial relationships of tissue invasion
Source: Acta Neuropathol Commun. 2016 Jun 3;4:57. doi: 10.1186/s40478-016-0321-8 (PMC4891921; doi:10.1186/s40478-016-0321-8)
Supplement: Additional file 1: Table S1. — Case Details. (DOC 27.5 kb) [file 40478_2016_321_MOESM1_ESM.doc]

Supplementary Table: Case Details:

| Case: | Age of Patient: | Type: | Comment: |
| --- | --- | --- | --- |
| 1 | 10 years | Primary resection | Radiological Hypothalamic damage prior to surgery. At follow up panhypopituitarism requiring hormonal replacement |
| 2 | 8 years | Primary resection | Clinical details not available. |
| 3 | 43years | Primary resection | Imaging at diagnosis shows calcifed lesion impinging on 3^rd^ ventricle. At follow up panhypopituitarism and obesity. |
